# Supplementary material for: Impact of Pacific Ocean heatwaves on phytoplankton community composition
Source: Commun Biol. 2023 Mar 13;6:263. doi: 10.1038/s42003-023-04645-0 (PMC10008836; doi:10.1038/s42003-023-04645-0)
Supplement: Supplementary file 2 — Supplementary Material [file 42003_2023_4645_MOESM2_ESM.pdf]

Supplementary Information for

Impact of Pacific Ocean heatwaves on phytoplankton community composition

Lionel A. Arteaga[1,2*], Cecile S. Rousseaux [3]

(*) Corresponding author

Afiliations:

- (1) Global Modeling and Assimilation Office, NASA Goddard Space Flight Center, Greenbelt, MD 20771, USA
- (2) Goddard Earth Sciences, Technology and Research II, University of Maryland Baltimore County, Baltimore, MD 21250, USA
- (3) Ocean Ecology Laboratory, NASA Goddard Space Flight Center, Greenbelt, MD 20771, USA

Supplementary note 1

General PFT description

In the present study, an ocean biogeochemical model with explicit representation of six different phytoplankton functional types (PFTs) was employed in order to compute temporal anomalies in their Chl-based biomass and assess the relative contribution of each group to the total surface Chl concentration. Diatoms represent a group of fast-growing phytoplankton with demand for high nutrient concentrations and characterized by the formation of an outer silica shell that enhances their sinking towards greater depths. Cyanobacteria represent a functional opposite, with slow division and sinking rates, and low nutrient requirements. Coccolithophores depict an intermediate group with moderate nutrient demands and growth

rates, relatively low light requirements, and relatively high sinking rates due to their calcium carbonate shell (coccoliths). Chlorophytes encompass a wide range of characteristics associated with nanoplankton, with intermediate growth and sinking rates as well as nutrient requirements. Dinoflagellates are similar to diatoms in their fast growth and high nutrient demands, but are distinguished by very high light requirements and negligible sinking rates. Finally, phaeocystis are also characterized by relatively high light requirements and sinking rates, but present slow division rates and low nutrient requirements (Table S1 and S2).

Climatological patterns

The mean surface distribution of the main nutrients determining phytoplankton growth and the six PFTs represented in the model were computed for the entire analyzed time span (2002–2020) (Figure S1). The mean distribution of the different phytoplankton groups is in accordance with their division rate speed as well as nutrient and light requirements, where diatoms dominate in high nutrient areas and cyanobacteria dominate in oligotrophic regions. Coccolithophores occupy transition regions in higher latitudes with seasonal light levels, while chlorophytes are mostly distributed along the margins between high and low nutrient conditions in the tropical Equatorial Pacific. Dinoflagellates occupy the largest portion of Chl biomass near the coast in the Gulf of Alaska and along the California Current System (CCS), in the ARC region, and towards the western part of the North Pacific. Phaeocystis are only relatively dominant in the western sub-arctic Pacific.

Anomalies in relative (%) phytoplankton biomass change

Maps of anomalies in phytoplankton biomass for all PFTs groups are calculated in relative percentage (%) during periods of noticeable change in phytoplankton community composition in GOA (May – December, 2014) and ENSO 3.4 (November 2015 – March 2016) (Figure

S2 and S3). All groups show noticeable changes in relative biomass. Nevertheless, anomalies in relative biomass can be negligible when converted to chlorophyll concentrations units (mg m^{-3}) and compared to changes in total bulk surface chlorophyll. This is the case in GOA for cyanobacteria, chlorophytes and phaeocystis (Figure 5 and S2), and in ENSO 3.4 for dinoflagellates and phaeocystis (Figure 6 and S3).

Anomalies in MLD and light limitation

We examined the relationship between anomalies in nutrients, temperature, and mixed layer depth at GOA and ENSO 3.4 (Figure S4). In GOA, shoaling of the oceanic surface mixed layer is associated with reduced silicate concentration and warmer temperatures (Figure S4a). In ENSO 3.4, shallower mixed layer depths are instead associated with cool SST anomalies and high nitrate concentrations (Figure S4b). Positive anomalies in the light growth-limiting term indicate that light limitation was reduced at GOA (Figure S5a) during the expansion of the Blob due to shoaling of the upper mixed layer. At ENSO 3.4, light limitation was reduced (i.e., positive anomalies in the light growth-limiting term) (Figure S5b) due to a strong decline in phytoplankton chlorophyll during the 2016 El Niño, leading to an overall diminishment in light attenuation.

Model validation

Chlorophyll

Temporal anomalies in total Chl from the NOBM are compared with satellite-based anomalies obtained from MODIS and VIIRS (Figure S6). This comparison is obtained by matching the spatial resolution of the NOBM output to that of the satellite data and masking model output in accordance with gaps in the satellite retrievals. Overall, the temporal anomaly in total Chl obtained from the NOBM is similar to that inferred from MODIS and VIIRS

retrievals. The skill of the model in reproducing surface satellite-based chlorophyll concentration is evaluated against global MODIS data by calculating the annual percent error (PE) for years where MODIS data for all 12 months are available (2003–2020) (Figure S8). Globally, the PE of the model is within ± 9 % of MODIS retrievals. For the North Pacific and North Central Pacific the model PE is within ± 15 %, while the model chlorophyll PE for the Equatorial Pacific basin is within ± 12 % (Figure S8) (see pre-defined model validation basins in Figure S7).

Phytoplankton Functional Types

In situ climatological observations are used to evaluate the skill of the model in reproducing the relative abundance of PFT with respect to total surface chlorophyll (Table S3). In situ climatological data allows for the assessment of modeled diatoms, coccolithophores, chlorophytes, cyanobacteria and phaeocystis. Globally, the largest difference in estimated relative abundance by the model is of -14.48 % for cyanobacteria, while other groups are within ± 10 %. In the North Pacific (NP), the relative abundance of diatoms and coccolithophores is overestimated in the model by (+) 30.62 % and (+) 41.18 %, respectively. The difference between modeled and in situ climatological relative abundance is much reduced for cyanobacteria (-1.03 %) and phaeocystis (-1.2 %). In the equatorial Pacific (EP), the largest difference in model-based relative abundances is of +30.9 % for chlorophytes, and -26 % for cyanobacteria, while diatoms and coccolithophores are within ± 3 %.

Nutrients

Modeled nutrient fields are validated against the observational datasets described in the Methods section[1,2] (by computing the PE, similarly as for chlorophyll). Globally, the model underestimates the surface (mean mixed layer) concentration of in situ silica by about (-) 46 %, while the observed mean nitrate and iron concentration are underestimated by ∼ (-)

24 % (Figure S9). In the North Pacific, the largest disparity is obtained in modeled silica, which underestimates in situ fields by about (-) 56 %. Modeled surface nitrate is slightly lower than observations (-3 %), which represents an important improvement with respect to previous versions of the NOBM where in situ nitrate was overestimated by a factor of about 1.5[3]. Observed dissolved iron in this region is underestimated by a similar low relative amount (-3 %). For both the North Central Pacific (NCP) and Equatorial Pacific (EP) basins, modeled iron shows the largest disagreement with observations in the order of -66 % and +55 %, respectively. Observed silica is underestimated in the NCP by (-) 45 %, but overestimated in the EP by $\sim$ (+) 40 %. In both of these regions, modeled nitrate has the lowest discrepancy with respect to in situ observations, with an overestimation of about (+) 15 % (Figure S9).

Table S1: Parameter values of phytoplankton maximum growth (division) rate (μ_{max}), sinking rate at 31°C (w_0), and half saturation concentration for nitrate ($k_{\mathrm{NO_3}}$), iron (k_{Fe}), and silica (k_{Si}), set for each PFT in the NOBM. The k value for ammonium is the same as for nitrate.

PFT	μ_{max} (d^{-1})	w_0 (m d^{-1})	$k_{\mathrm{NO_3}}$ (μmol kg^{-1})	k_{Fe} (nmol kg^{-1})	k_{Si} (μmol kg^{-1})
Diatoms	2.75	0.75	1	0.12	0.2
Coccolithophores	2.14	0.65	0.5	0.8	-
Chlorophytes	2.35	0.25	0.67	0.09	-
Dinoflagellates	2.48	0	1	0.12	-
Cyanobacteria	1.84	8.5e-3	0.45	0.8	-
Phaeocystis	1.38	0.75	0.1	0.24	-

Table S2: Parameter value of the half light saturation parameter (k_E) (μE m^{-2} d^{-1}) set for each PFT in the NOBM. The NOBM employs three different light level thresholds to determine k_E: $<$ 50 μE m^{-2} d^{-1} (low), $\geq$ 50 and $\leq$ 200 μE m^{-2} d^{-1} (medium), $>$ 200 μE m^{-2} d^{-1} (high).

PFT	k_E (low)	k_E (medium)	k_E (high)
Diatoms	30	93	184
Coccolithophores	19	71	165
Chlorophytes	32	87	144
Dinoflagellates	40	128	270
Cyanobacteria	22	66	47
Phaeocystis	67	117	196

Table S3: Difference in mean relative abundance of each PFT Chl biomass to total Chl (%) between model and climatological observations ($PFT\%_{model} - PFT\%_{obs}$), computed for the last year of NOBM output (2020). Missing data (-) indicates unavailable climatological observations. In situ data on dinoflagellates are insufficient to evaluate model output. Regions evaluated are the North Pacific (NP), Equatorial Pacific (EP), North Central Pacific (NCP), South Pacific (SP), North Atlantic (NA), Equatorial Atlantic (EA), North Central Atlantic (NCA), South Atlantic (SA), North Indian (NI), Equatorial Indian (EI), South Indian (SI), Antarctic (ANT).

Region	Diatoms	Coccolithophores	Chlorophytes	Cyanobacteria	Phaeocystis
Global	8.42	9.93	-2.85	-14.48	-0.22
NP	30.62	41.18	-	-1.03	-1.2
EP	-2.67	1.35	30.9	-26	-
NCP	6.11	-20.8	-0.29	8.91	-
SP	25.96	-10.98	-21.22	-10.22	-
NA	-2.87	55.05	-40	-5.15	-0.84
EA	3.16	-	-	-28.38	-
NCA	7.13	-5.54	4.51	4.81	-
SA	15.83	-39.93	-43.74	18.15	-
NI	23.12	-5.21	18.71	-29.15	-
EI	2.54	-	69.94	-52.48	-
SI	-	-	-	-	-
ANT	-16.35	-5.57	-44.5	-2.4	1.38

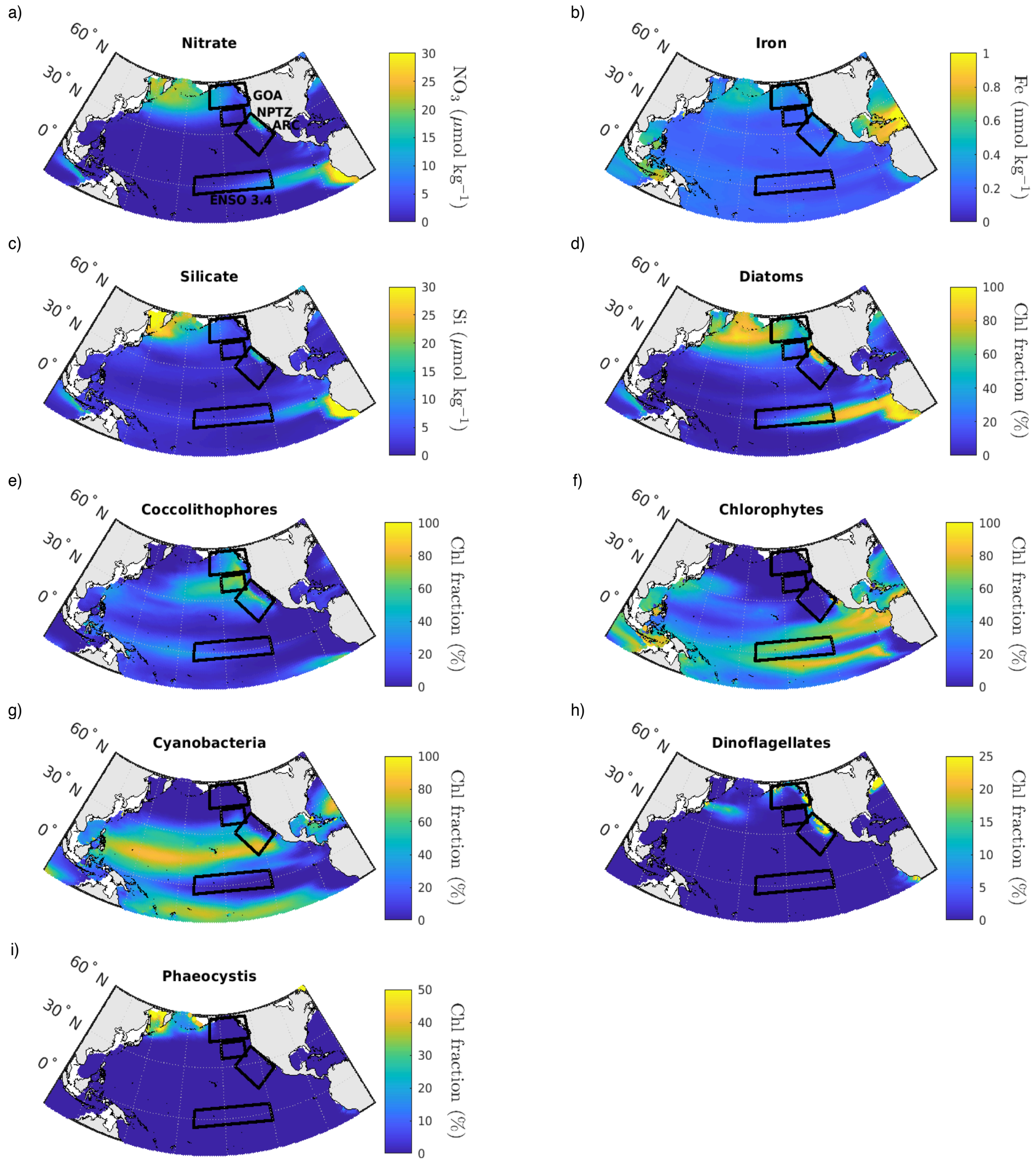

Figure S1: Mean biogeochemical output from the NOBM for the surface mixed layer between 2002 and 2020: (a) Nitrate, (b) iron, (c) silicate, (d) diatoms, (e) coccolithophores, (f) chlorophytes, (g) cyanobacteria, (h) dinoflagellates, and (i) phaeocystis.

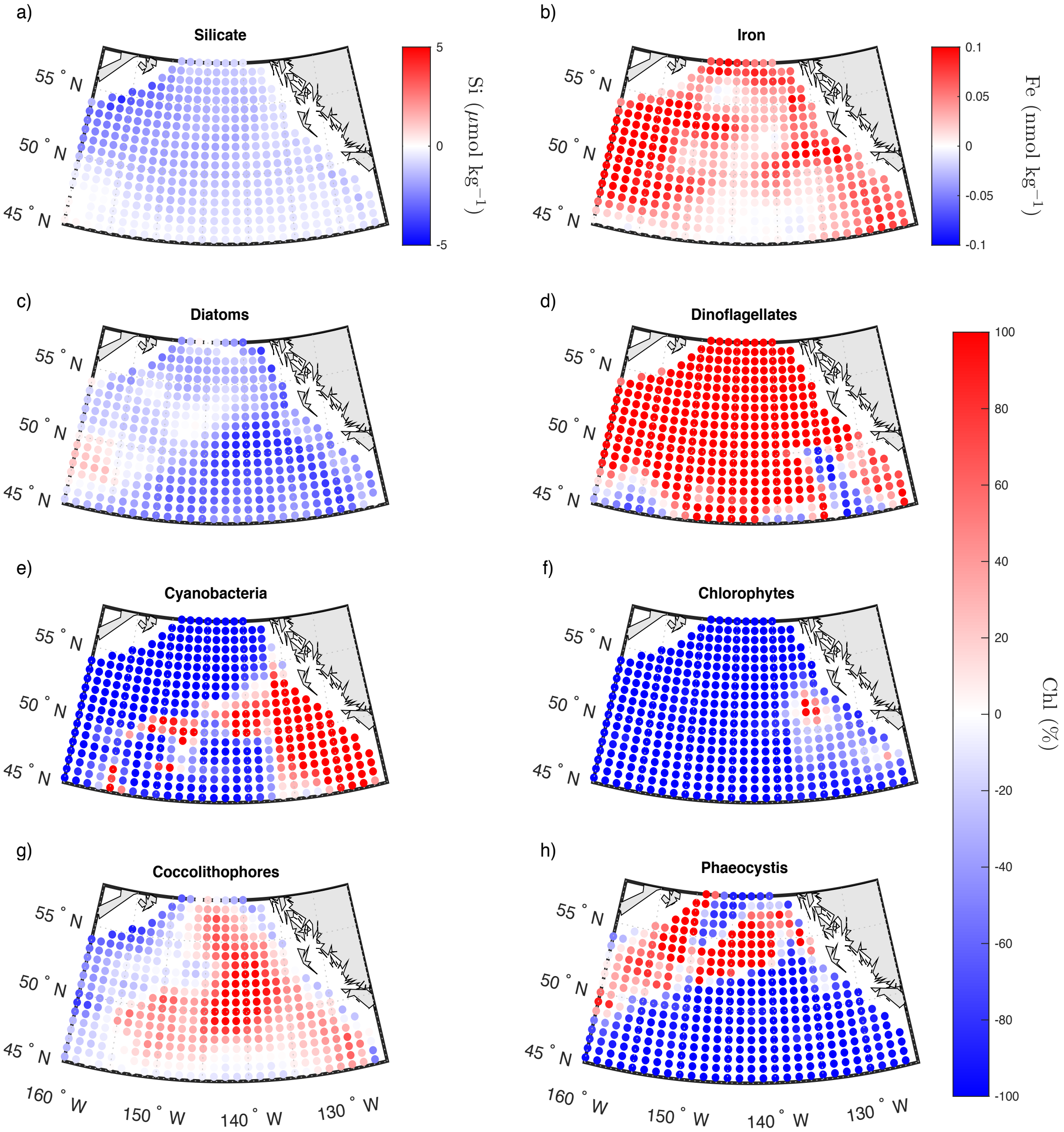

Figure S2: Spatial pattern in GOA nutrient concentration anomalies and PFTs relative anomalies between May and December of 2014. a & b) Mean anomaly in modeled surface (a) silicate (μmol kg^{-1}) and (b) iron (nmol kg^{-1}). c–h) Mean anomaly in the modeled surface chlorophyll biomass (%) of (c) diatoms, (d) dinoflagellates, (e) cyanobacteria, (f) chlorophytes, (g) coccolithophores, and (h) phaeocystis. Panels (a) and (b) are the same as in Figure 5, and are shown here for reference.

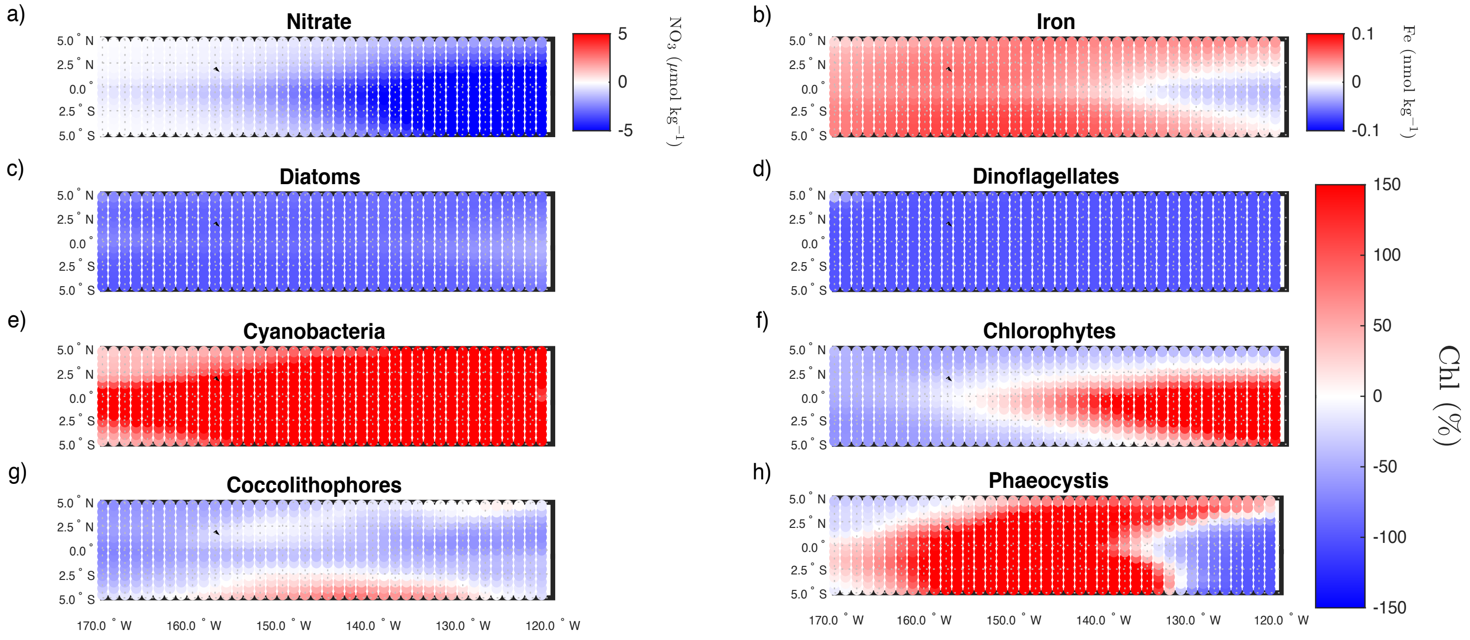

Figure S3: Spatial pattern in ENSO 3.4 nutrient concentration anomalies and PFTs relative anomalies between November of 2015 and March of 2016. a & b) Mean anomaly in modeled surface (a) nitrate (μmol kg^{-1}) and (b) iron (nmol kg^{-1}). c–h) Mean anomaly in the modeled surface chlorophyll biomass (%) of (c) diatoms, (d) dinoflagellates, (e) cyanobacteria, (f) chlorophytes, (g) coccolithophores, and (h) phaeocystis. Panels (a) and (b) are the same as in Figure 6, and are shown here for reference.

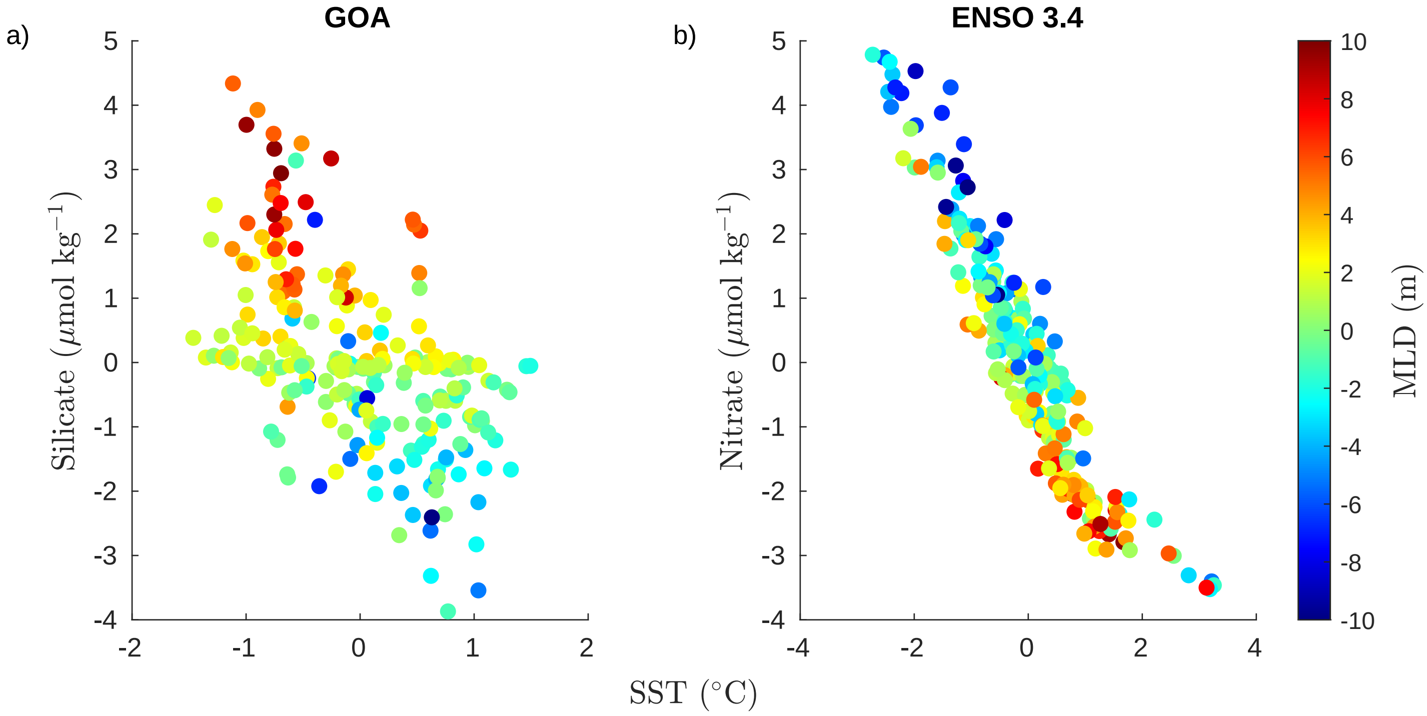

Figure S4: Scatterplot of anomalies in (a) modeled Sea Surface Temperature (SST) (°C), surface silicate (μmol kg^{-1}), and mixed layer depth (MLD) (m) in GOA, and (b) anomalies in modeled SST, surface nitrate (μmol kg^{-1}), and MLD in the ENSO 3.4 region. Positive and negative anomalies in MLD indicate deepening and shoaling of the surface mixed layer, respectively.

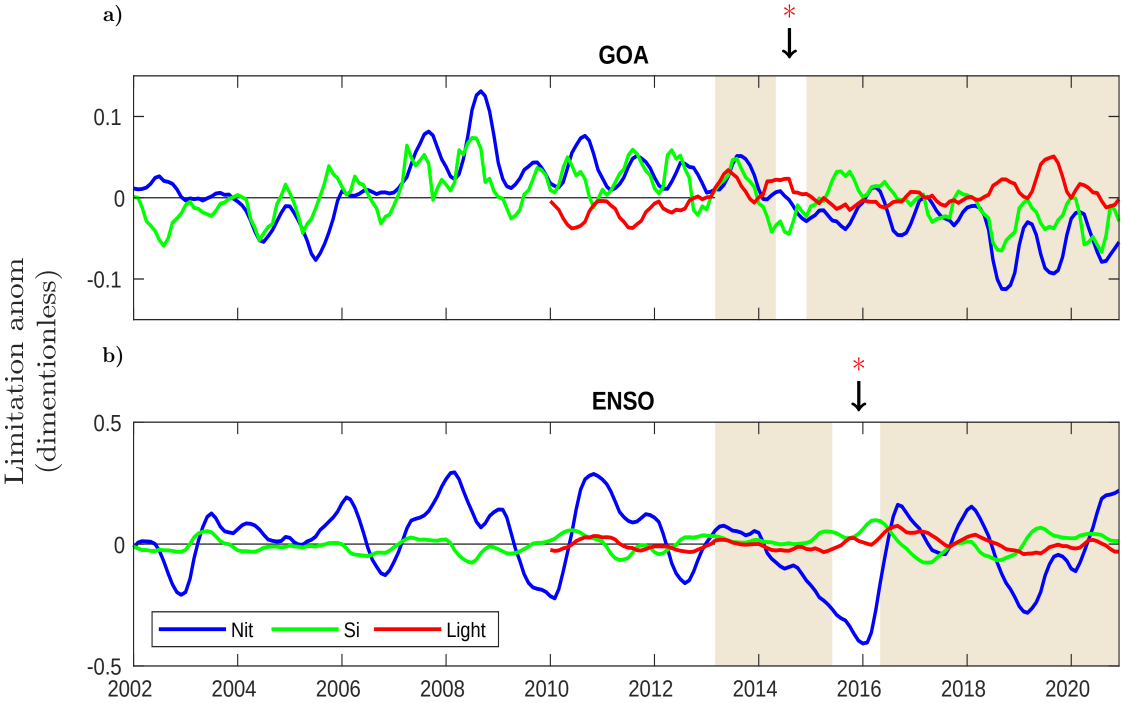

Figure S5: Time series of monthly anomalies in the mixed layer growth-limiting term of nitrate (blue line), silicate (green line), and light (red line) for (a) GOA and the (b) ENSO 3.4 region. Similar as in Figure 2a and 3b, red asterisks represent the periods where notorious shifts in PFTs relative composition were found in GOA (May–December 2014) and the ENSO 3.4 region (November 2015–March 2016).

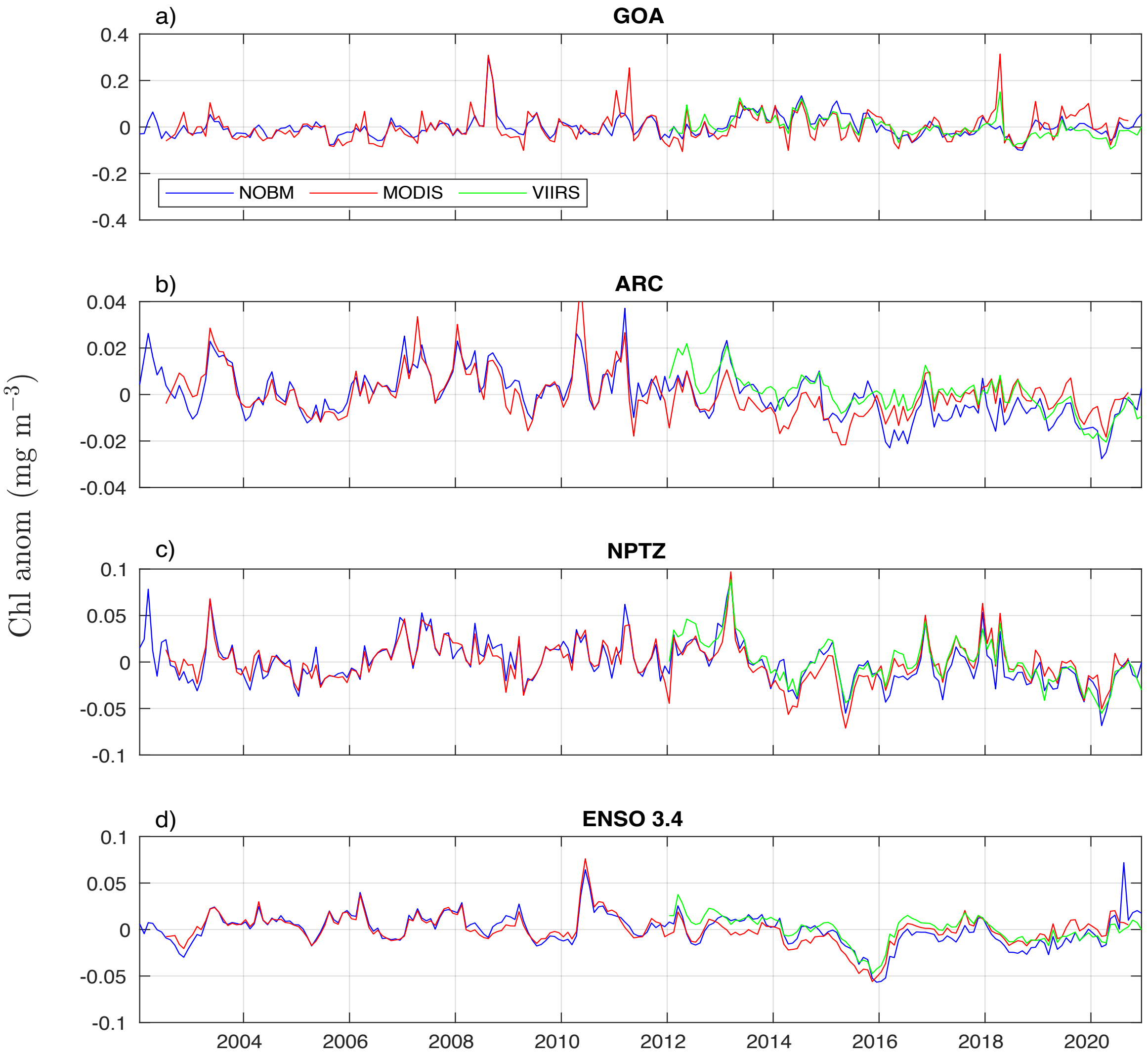

Figure S6: Time series of monthly surface chlorophyll anomalies from the NOBM (blue line), MODIS (red line) and VIIRS (green line), in the four evaluated regions: (a) GOA, (b) ARC, (c) NPTZ, and (d) ENSO 3.4.

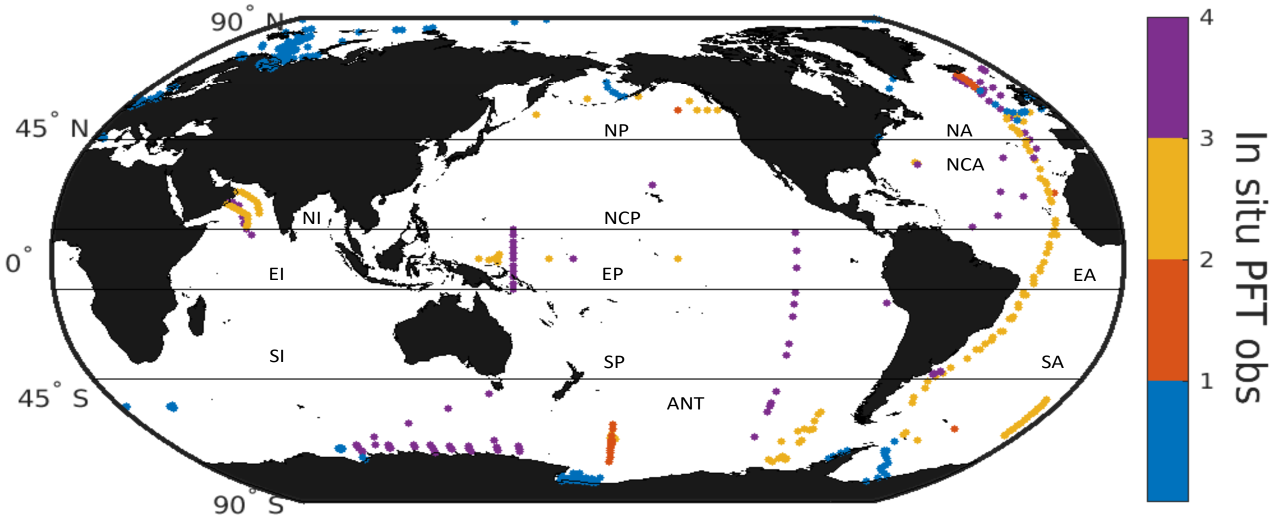

Figure S7: Distribution of in situ observations of PFTs relative abundances (i.e., percentage of Chl-based biomass relative to total chlorophyll) used for validation of the NOBM. Color scale indicates the total number of functional types observed at each location (out of the six PFTs represented in the model).

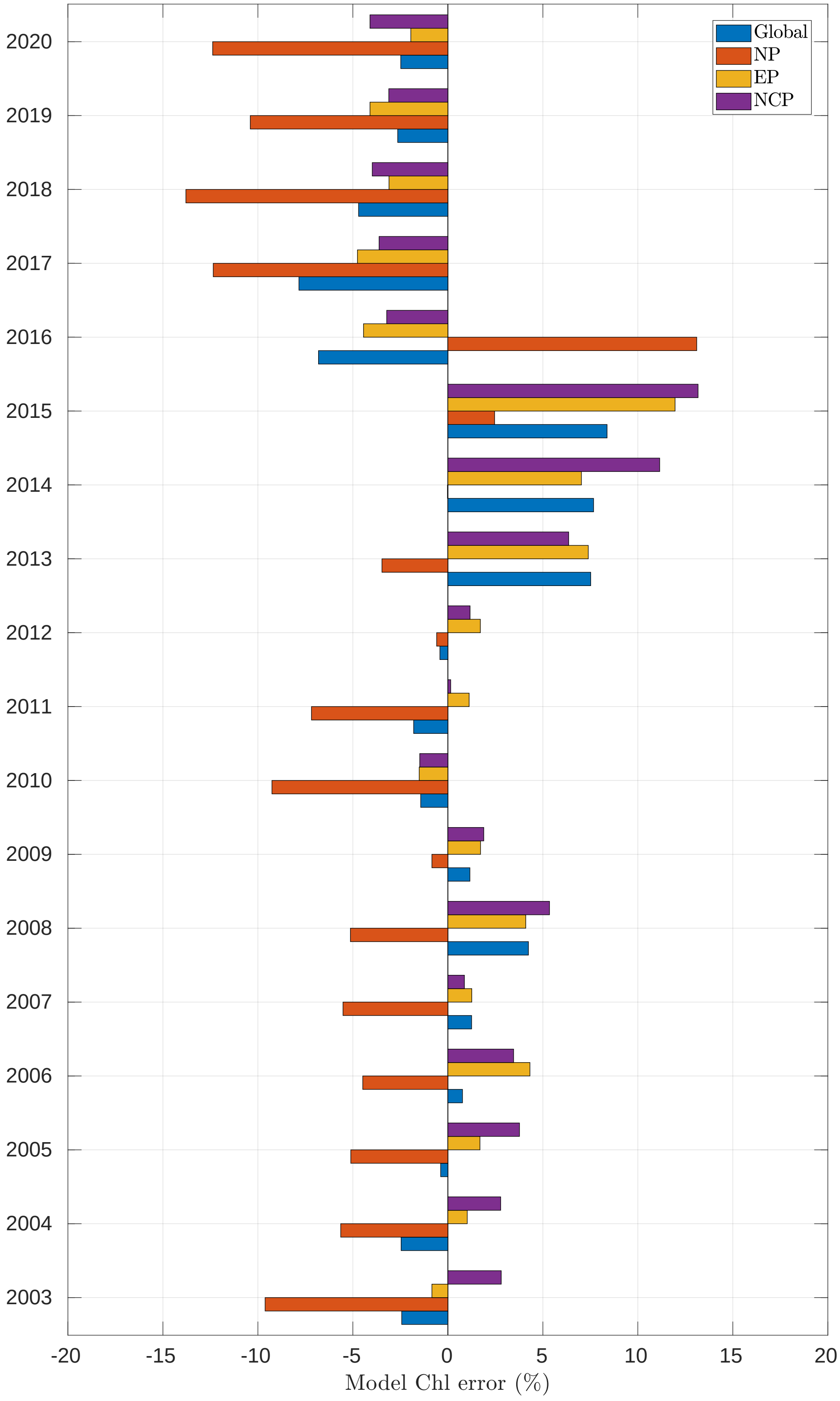
Global
NP
EP
NCP
2020
2019
2018
2017
2016
2015
2014
2013
2012
2011
2010
2009
2008
2007
2006
2005
2004
2003
-20
-15
-10
-5
0
5
10
15
20
Model Chl error (%)

Figure S8: (Previous page.) Annual percent error (PE) (%) of the NOBM surface chlorophyll output compared against MODIS data (Equation 9) computed over the global domain (Global), the North Pacific (NP), North Central Pacific (NCP), and Equatorial Pacific (EP) basins (see Figure S7 for basins boundaries). PE is not computed for year 2002, as monthly MODIS data for that year is incomplete (MODIS output is available from July 2002 onwards).

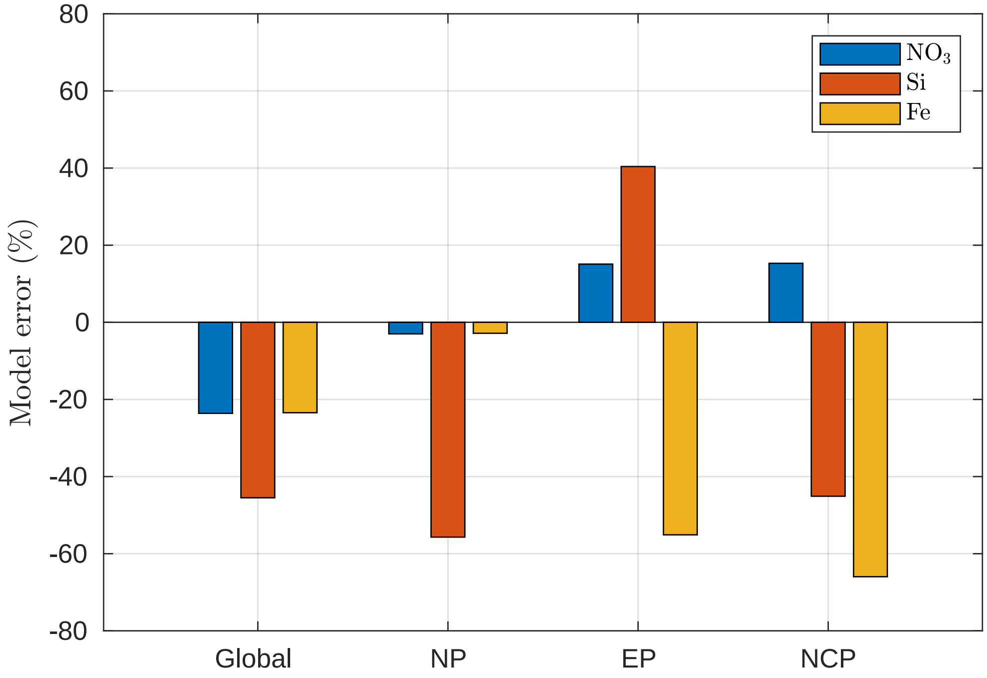

Figure S9: Annual percent error (PE) in modeled surface (upper mixed layer) nutrient fields (nitrate, silicate, and dissolved iron) for the last year of NOBM output (2020) compared against in situ observations globally and within the North Pacific (NP), North Central Pacific (NCP), and Equatorial Pacific (EP) basins (see Figure S7 for basins boundaries).

References

1. Conkright, M. E. *et al.* World Ocean Atlas 2001. Volume 4, Nutrients. In *NOAA atlas NESDIS ; 52*, vol. 4, 392 (US Government Printing Office, Washington, DC, 2002). URL `https://repository.library.noaa.gov/view/noaa/1102`.

2. Gregg, W. W., Ginoux, P., Schopf, P. S. & Casey, N. W. Phytoplankton and iron: validation of a global three-dimensional ocean biogeochemical model. *Deep Sea Research Part II: Topical Studies in Oceanography* **50**, 3143–3169 (2003). URL `https://www.sciencedirect.com/science/article/pii/S096706450300184X`. The US JGOFS Synthesis and Modeling Project: Phase II.

3. Gregg, W. W. & Casey, N. W. Modeling coccolithophores in the global oceans. *Deep Sea Research Part II: Topical Studies in Oceanography* **54**, 447–477 (2007). URL `https://www.sciencedirect.com/science/article/pii/S0967064507000318`. The Role of Marine Organic Carbon and Calcite Fluxes in Driving Global Climate Change, Past and Future.
